# Identification of Antigenic Proteins from *Lichtheimia corymbifera* for Farmer’s Lung Disease Diagnosis

**DOI:** 10.1371/journal.pone.0160888

**Published:** 2016-08-04

**Authors:** Bénédicte Rognon, Coralie Barrera, Michel Monod, Benoit Valot, Sandrine Roussel, Manfredo Quadroni, Stephane Jouneau, Isabelle Court-Fortune, Denis Caillaud, Jean-Marc Fellrath, Jean-Charles Dalphin, Gabriel Reboux, Laurence Millon

**Affiliations:** 1 UMR6249 Chrono-Environnement, University of Bourgogne Franche-Comté, Besançon, France; 2 Parasitology-Mycology Department, University Hospital of Besançon, Besançon, France; 3 Department of Dermatology, University Hospital, Lausanne, Switzerland; 4 Protein Analysis Facility, Center for Integrative Genomics, University of Lausanne, Lausanne, Switzerland; 5 Pneumology Department, University Hospital of Rennes, Rennes, France; 6 UMR1085, IRSET, Rennes 1 University, Rennes, France; 7 Pneumology Department, University Hospital North, Saint-Etienne, France; 8 Pneumology Department, University Hospital Gabriel Montpied, Clermont-Ferrand, France; 9 Department of Pulmonary Medicine, Pourtalès Hospital, Neuchatel, Switzerland; 10 Department of Respiratory Disease, University Hospital of Besançon, Besançon, France; Leibniz-Institut fur Naturstoff-Forschung und Infektionsbiologie eV Hans-Knoll-Institut, GERMANY

## Abstract

The use of recombinant antigens has been shown to improve both the sensitivity and the standardization of the serological diagnosis of Farmer’s lung disease (FLD). The aim of this study was to complete the panel of recombinant antigens available for FLD serodiagnosis with antigens of *Lichtheimia corymbifera*, known to be involved in FLD. *L*. *corymbifera* proteins were thus separated by 2D electrophoresis and subjected to western blotting with sera from 7 patients with FLD and 9 healthy exposed controls (HEC). FLD-associated immunoreactive proteins were identified by mass spectrometry based on a protein database specifically created for this study and subsequently produced as recombinant antigens. The ability of recombinant antigens to discriminate patients with FLD from controls was assessed by ELISA performed with sera from FLD patients (n = 41) and controls (n = 43) recruited from five university hospital pneumology departments of France and Switzerland. Forty-one FLD-associated immunoreactive proteins from *L*. *corymbifera* were identified. Six of them were produced as recombinant antigens. With a sensitivity and specificity of 81.4 and 77.3% respectively, dihydrolipoyl dehydrogenase was the most effective antigen for discriminating FLD patients from HEC. ELISA performed with the putative proteasome subunit alpha type as an antigen was especially specific (88.6%) and could thus be used for FLD confirmation. The production of recombinant antigens from *L*. *corymbifera* represents an additional step towards the development of a standardized ELISA kit for FLD diagnosis.

## Introduction

Hypersensitivity pneumonitis (HP) is an inflammatory lung disease that is caused by an exacerbated immune response to repeated inhalation of antigens [1]. Farmer’s lung disease (FLD), one of the most common occupational HP in the world [2], results from the chronic inhalation of antigens released into the farmer’s environment by microorganisms, especially during the handling of spoiled hay or straw. The main recognized causative agents of FLD are actinomycetes such as *Sacchropolyspora rectivirgula*, and molds of various genera including Aspergillus species and *Lichtheimia corymbifera* [3–7]. The role in FLD of the mucoraceous mold *L*. *corymbifera* (formerly *Absidia corymbifera)* is supported by several studies demonstrating i) high levels of specific precipitins of *L*. *corymbifera* in FLD patients’ sera [3,6], ii) FLD relapses due to high amounts of *L*. *corymbifera* in FLD patient-handled hay [8], and iii) a marked inflammatory response of pulmonary epithelial cells exposed to *L*. *corymbifera* [9].

Over the past few decades, HP immunodiagnosis has been based on the use of crude fractions of the microorganisms involved in occupational exposure [10,11]. However, the current use of recombinant antigens improves both the sensitivity and the standardization of serodiagnosis [12]. A strategy consisting in using sera from patients with FLD to identify immunogenic proteins from environmental microorganisms was applied and allowed the selection of immunoreactive proteins from *S*. *rectivirgula* [13] and *Aspergillus* species [14]. Recombinant antigens from these two species were produced and proved to be effective in the serodiagnosis of FLD by enzyme-linked immunosorbent assay (ELISA). A panel including *L*. *corymbifera* recombinant antigens was expected to further improve the diagnostic performance of the ELISA test. Comparative western blotting (WB) analysis, performed with *L*. *corymbifera* protein extract and sera from patients with FLD and healthy exposed controls (HEC), identified bands specific to FLD at 27.7, 40.3, 44.0, and 50.5 kDa [15]. Proteins included in these bands might be suitable candidates.

Testing recombinant antigens issued from three microorganisms is necessary due to the microbiological evolution of hay stocks over the course of a year. Farmers are successively exposed, and for variable lengths of time, to the microflora of the field, and to that of both hay storage and decayed hay. First, thermotolerant fungi (that is *Eurotium amstelodami* or *L*. *corymbifera*) develop and cause biochemical changes and an increase in temperature which provide the optimum conditions for *S*. *rectivirgula* and other thermophilic actinomyces to grow [16]. At each step, except under field microflora exposure, cases of farmer’s lung can appear. Consequently, some cases are sensitive to one, two or three of these microorganisms.

The main objective of the present study was to identify immunoreactive proteins of *L*. *corymbifera* associated to FLD status so as to complete the panel of recombinant antigens useful for a standardized and sensitive serodiagnosis of the disease.

## Materials and Methods

### Patients

Patients (*n* = 41) diagnosed with FLD pulmonary syndrome on the basis of consensus criteria [17,18] were consecutively recruited from the pneumology departments of five university hospitals in France and Switzerland from 2006 to 2012. Patient characteristics were described in detail in a previous study [13]. HEC (*n* = 43) were recruited from the same areas. The protocol was approved by the local ethics committee (*Comité de Protection des Personnes EST-II*–Regional Health Agency of Franche-Comté (*Agence Régionale de Santé de Franche-Comté*, France)), and a written informed consent was obtained from each participant (*Projet Hospitalier de Recherche Clinique* “SOPHIA” 2009-A00546-51). Blood samples were taken from each individual and centrifuged within 4 hours of sampling. Sera were immediately frozen and stored at -80°C until use.

Stored sera from 13 patients diagnosed with probable or proven mucormycosis between 2004 and 2013 were also analyzed using ELISA with *L*. *corymbifera* recombinant antigens. Stored sera from immunocompromised patients without invasive fungal infection (n = 8) and sera from healthy subjects (n = 16) were also tested by the same ELISA test.

### Identification of immunoreactive proteins

The immunoproteomic approach previously described for identification of immunoreactive proteins from *S*. *rectivirgula* [13] was used in this study with only a few changes. Protein extracts from *L*. *corymbifera* (reference strain BBCM/IHEM 3809 isolated from FLD-linked hay) were prepared as previously described [15]. Briefly, the process consisted of the enzymatic lysis of the fungal cell wall with a recombinant β-1,3-glucanase (Lyticase from *Arthrobacter luteus*, Sigma-Aldrich^®^, St Louis, MO, USA), followed by protein precipitation with trichloroacetic acid in presence of deoxycholate and protein purification with the SDS-Page Clean-Up Kit (GE Healthcare^®^, Piscataway, USA). A set of sera from FLD patients (n = 7) and from HEC (n = 9) which detect bands representative of each group with the weakest background signals was selected from a previous 1D-WB experiment [15]. Protein extracts (25 μg) were separated by two dimensional SDS-PAGE on small size gels, and immunoreactive proteins were detected by WB using the selected sera. Comparing blots allowed us to identify spots specifically detected with patient sera. A large size gel and membrane were then prepared with 50 and 100 μg of protein extract, respectively, to locate the spots of interest with greater accuracy. Spots of interest were excised from the large 2D gel colored with colloidal Coomassie blue after alignment with the large membrane probed with four representative sera from FLD patients. Corresponding proteins, putative markers of the disease, were then identified by liquid chromatography coupled with tandem mass spectrometry (LC-MS/MS) analysis. Sequencing of *L*. *corymbifera* mRNA was performed to create a database for protein identification by mass spectrometry, because at the beginning of this study, the *L*. *corymbifera* genome had not yet been sequenced.

### *L*. *corymbifera* RNA extraction, mRNA sequencing and database construction

*L*. *corymbifera* RNA was extracted from cells grown on DG18 agar medium for one week at 30°C. Fungal cells from 20 plates were then resuspended in 60 ml of sodium phosphate buffer (10 mM Na_3_PO_4_ and 0.15 M NaCl, pH 6). After 10 min of centrifugation at 12,000 x g at 4°C, pelleted cells were frozen in liquid nitrogen to disrupt the fungal cell wall, and total RNA was extracted using the RNeasy Mini kit (QIAgen^®^, Hilden, Germany), according to the manufacturer’s instructions.

Shotgun sequencing of normalized mRNA sample was performed by Microsynth (Balgach, Switzerland) using 454 FLX technology (454 Roche GS FLX, Roche^®^), and contigs were assembled with the GS De Novo Assembler version 2.5.3 using the default settings for cDNA. This run generated 269,021 reads with an average length of 323.3 bases, and 11,529 contigs (average length of 810 bases) were reconstructed. 7,110 were qualified as large contigs (>500 bases).

A single file in FASTA format compiling all amino acid sequences derived from the translation of these contigs according to the three possible reading frames was used as a database for protein identification by mass spectrometry.

During the preparation of this manuscript, the *L*. *corymbifera* genome (strain FSU 9682, CBS 429.75, ATCC 46771) was sequenced, and annotated protein sequences were added to the National Center for Biotechnology (NCBI) database by Schwartze et al. [19]. A blastp with ncbi databases was performed to annotate the 41 proteins (potential diagnostic markers) identified in this study by mass spectrometry based on our database. Thirty-three out of the 41 predicted amino acid sequences were 98 to 100% identical to the *L*. *corymbifera* newly published protein sequences. The eight remaining sequences aligned with those of *Absidia idahoensis var*. *thermophila*, with identity percentages comprised between 84 and 99%.

### Development of recombinant antigens

Open reading frames of proteins of interest, deduced from *L*. *corymbifera* mRNA sequencing, were chemically synthesized, inserted between the *Nco*I and *Bam*HI restriction sites of the expression vector pET-11aH6 [20] by the Genecust laboratory (Luxembourg). After cloning in *Escherichia coli* strain DH5α, recombinant plasmids were isolated and then inserted into bacterial strain BL21 for protein expression. Recombinant protein production was induced by adding isopropyl β-D-1-thiogalactopyranoside (final concentration 0.1 mM) for 4 hours to mid-log phase cells cultured in LB medium, and 6 × His-tagged proteins were extracted by affinity chromatography on Ni-NTA resin (Qiagen^®^) columns as recommended by the manufacturer.

### ELISA tests

Detection of specific IgG in sera was performed by ELISA tests as described by Barrera et al. [13] using each of the six recombinant antigens produced.

Serum samples from patients with FLD (n = 41) and controls (n = 43), diluted 100 X, were deposited, in triplicate, in the wells of 96-well plates previously coated with 200 μl of recombinant antigen solution (1 μg/ml). Each plate included a reference sample (pool of sera from two FLD patients: FLD07 and FLD35) which was used for normalization between plates calculating ELISA indexes. ELISA tests were performed in three independent experiments. For each experiment, the two closest absorbance (*A*_450 nm_) values obtained with each serum were averaged, and an index corresponding to the ratio between the mean *A* of the serum sample and the mean *A* of the reference sample was calculated. The mean of the three index values obtained in the three independent experiments was then calculated for each serum sample.

For each recombinant antigen, we assessed the performance of the ELISA tests for FLD diagnosis by analyzing the receiver operating characteristics (ROC) curve (Stata9). The threshold, which best discriminated patients from controls, was determined from the best percentage for correct classification, with the best combination of sensitivity and specificity.

## Results

### Detection of immunoreactive proteins by 2D PAGE and WB with sera from FLD patients and HEC

*L*. *corymbifera* protein extracts were subjected to 2D PAGE on small gels and WB. Sixteen small membranes were prepared, and probed with the nine selected sera from HEC and the seven selected sera from FLD patients. All in all, 64 clearly distinct spots were detected on the different membranes probed with FLD sera (data not shown). All these spots were located and numbered on each of the membranes. Twenty-three spots were thought to be associated with FLD patients because i) they were detected with at least three of the seven sera from patients with FLD and ii) they were detected at least twice more often with FLD sera than with HEC sera.

**Fig 1:**
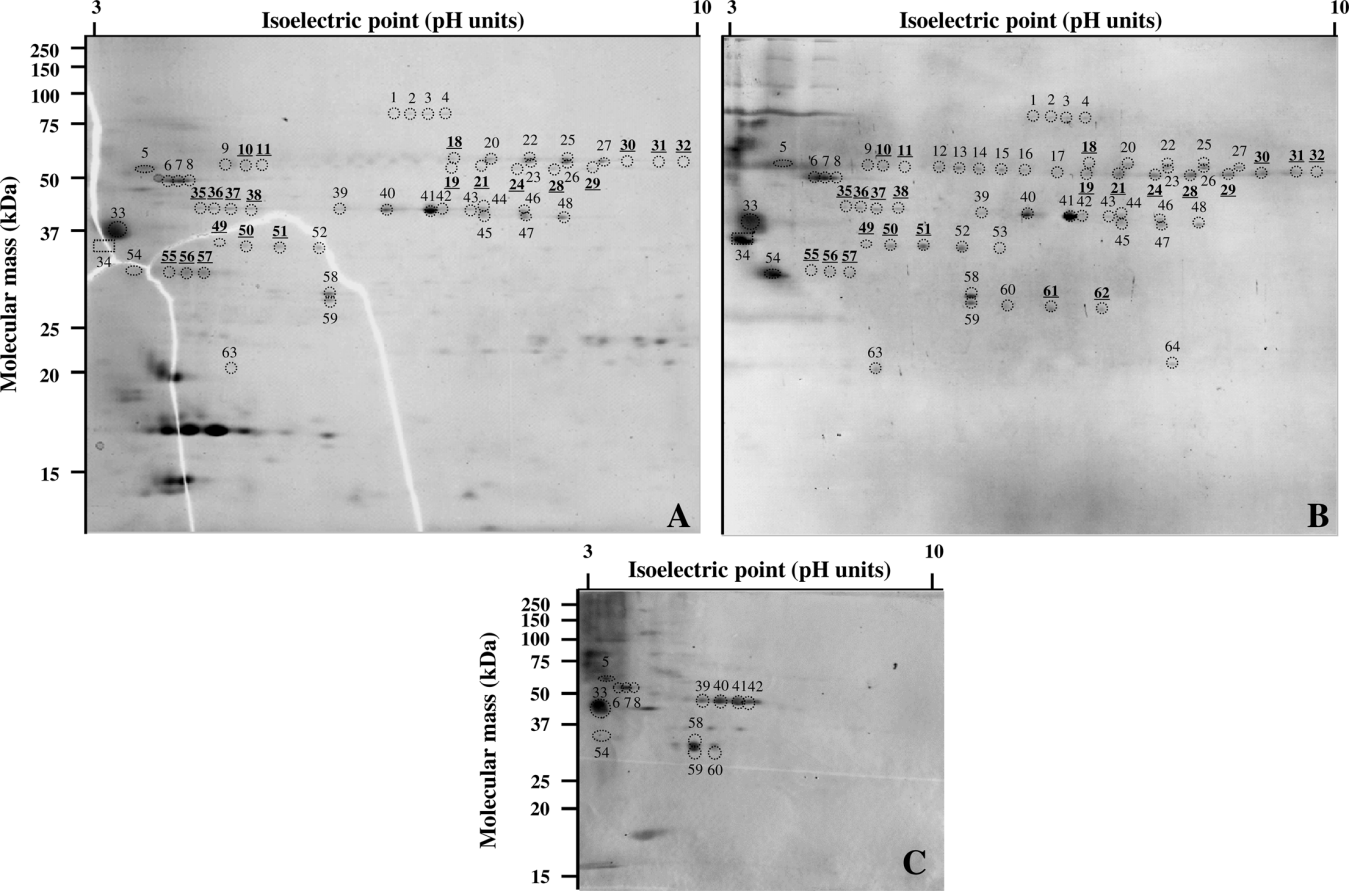
2D-electrophoresis of *L*. *corymbifera* protein extract and western blotting with sera from FLD patients and HEC. (A) Large gel stained with Coomassie blue. (B) Large membrane probed with a pool of representative sera from FLD patients (FLD02, FLD08, FLD10 and FLD12). (C) Small membrane probed with one serum representative of HEC (HEC22). Of the 64 immunoreactive spots detected with FLD sera and numbered on the large blot (B), only a few were recovered on each membrane probed with HEC sera (C). In contrast, 55 of these spots were clearly located on the gel colored with Coomassie blue (A). All spots defined as associated with FLD patients (spots with numbers written in boldface and underlined) were excised from the gel for protein identification by mass spectrometry, except spots 61 and 62 which were not accurately located on the gel.

We then carried out 2D PAGE on two large gels. The first gel was stained with Coomassie blue. About 200 clearly individualized spots could be seen on the gel (Fig 1A). Proteins from the second gel were transferred onto a large membrane that was probed with a pool of sera from four FLD patients (chosen among the 7 initially selected) which, when combined, allowed the detection of the 64 FLD spots (Fig 1B). Alignment between the gel and the membrane enabled the accurate location on the gel of 55 of the 64 spots previously seen using small membranes. Among the 23 spots described as associated with FLD status using small membranes, 21 were visible on the large gel and could be cut for LC-MS/MS analysis (Fig 1). Two spots (spots 61 and 62) were not visible on the gel.

Forty-one different proteins were identified from the excised spots. The characteristics and biological functions of these proteins are given in Table 1. Eleven proteins are responsible for energy production and conversion (LC1, LC3, LC5, LC8, LC10, LC19, LC20, LC22, LC35, LC36, LC39), eight are involved in carbohydrate transport (LC2, LC9, LC11, LC15, LC18, LC21, LC34, LC37), eight ensure posttranslational modifications and protein turnover (LC17, LC25, LC27-LC33), five play a role in amino acid transport and metabolism (LC6, LC7, LC14, LC24, LC26, LC35), and four are involved in lipid transport and metabolism (LC12, LC16, LC23, LC40). LC4 function consists of inorganic ion transport and metabolism. LC41 has a signal transducer activity.

**Table 1:** Characteristics of *L*. *corymbifera* immunoreactive proteins. Proteins written in bold were produced as recombinant antigens.

Code	ncbi accession number*	Protein identification	Biological function	Spot number on Fig 1 (spectra)^§^
LC1	CDH58998.1	Fumarate reductase	Energy production and conversion	28 (3); 29 (5)
**LC2**	CDH54616.1	**Pyruvate kinase**	Carbohydrate transport and metabolism	10 (5); 11 (3); 18 (6)
LC3	CDH52287.1	v-type atpase	Energy production and conversion	11 (5)
LC4	CDH60495.1	Catalase	Inorganic ion transport and metabolism	18 (4)
**LC5**	CDH55589.1	**Dihydrolipoyl dehydrogenase**	Energy production and conversion	10 (6); 11 (5); 18 (6)
LC6	CDH53460.1	Bleomycin hydrolase	Amino acid transport and metabolism	55 (2)
LC7	CDH59771.1	Carboxypeptidase y precursor	Amino acid transport and metabolism	55 (18); 56 (9); 57 (15)
LC8	CDH59240.1	Aldehyde dehydrogenase	Energy production and conversion	10 (3); 11 (3); 35 (2)
LC9	CDH56669.1	Enolase	Carbohydrate transport and metabolism	11 (2)
LC10	CDH49193.1	Atp synthase f1 beta subunit	Energy production and conversion	10 (3); 11 (3); 55 (2)
LC11	CDH58368.1	Carbohydrate esterase family 9 protein	Carbohydrate transport and metabolism	10 (2); 11 (2)
**LC12**	CDH58504.1	**Acyl oxidase**	Lipid transport and metabolism	35 (3); 36 (5); 37 (3); 38 (4)
LC13	CDH55682.1	Probable qcr2-40 kda ubiquinol cytochrome-creductase core protein 2	General function prediction only	35 (14); 36 (19); 37 (18); 38 (16)
LC14	*CDS05524*.*1*	Putative Glutamine synthetase	Amino acid transport and metabolism	55 (3); 56 (3); 57 (2)
LC15	CDH55124.1	Alpha-mannosidase	Carbohydrate transport and metabolism	36 (3)
LC16	*CDS10668*.*1*	Putative Isovaleryl-CoA dehydrogenase,mitochondrial	Lipid transport and metabolism	36 (2)
LC17	CDH49332.1	Heat shock protein hss1	Posttranslational modification, protein turnover, chaperones	55 (2)
LC18	*CDS11245*.*1*	Hypothetical protein LRAMOSA03508 (putative adenosine kinase)	Carbohydrate transport and metabolism	35 (3); 36 (2)
**LC19**	CDH52624.1	**Atp synthase subunit alpha**	Energy production and conversion	10 (3); 11 (5); 18 (6); 19 (8); 21 (9); 24 (13); 28 (12); 29 (11); 30 (9); 31 (13); 32(6)
**LC20**	CDH57483.1	**Malate dehydrogenase**	Energy production and conversion	35 (3); 49 (4); 50 (2); 51 (5); 55 (3); 57 (3)
LC21	CDH53020.1	Transaldolase	Carbohydrate transport and metabolism	49 (2)
LC22	*CDS13358*.*1*	Hypothetical protein LRAMOSA05536 (putative ATP synthase subunit gamma)	Energy production and conversion	55 (3); 56 (4); 57 (2)
LC23	CDH51333.1	Enoyl-hydratase	Lipid transport and metabolism	55 (6); 56 (3); 57 (2)
LC24	CDH59027.1	Saccharopine dehydrogenase	Amino acid transport and metabolism	10 (2); 11 (3)
LC25	*CDS10670*.*1*	Hypothetical protein LRAMOSA11156 (putative serine protease)	Posttranslational modification, protein turnover, chaperones	49 (2); 51 (2)
LC26	CDH60921.1	Glutamate dehydrogenase (nad ())	Amino acid transport and metabolism	10 (2); 11 (2)
LC27	CDH60383.1	Serine protease	Posttranslational modification, protein turnover, chaperones	35 (3); 36 (2); 49 (3); 50 (3); 51 (2)
**LC28**	*CDS13594*.*1*	**Putative proteasome subunit alpha type**	Posttranslational modification, protein turnover, chaperones	49 (2); 55 (5); 56 (3); 57 (2)
LC29	CDH55408.1	Proteasome subunit alpha type-6	Posttranslational modification, protein turnover, chaperones	57 (2)
LC30	CDH57557.1	20s proteasome subunit	Posttranslational modification, protein turnover, chaperones	55 (2)
LC31	CDH54172.1	N-terminal nucleophile aminohydrolase	Posttranslational modification, protein turnover, chaperones	55 (2)
LC32	CDH59238.1	Peptidase s28	Posttranslational modification, protein turnover, chaperones	11 (2)
LC33	*CDS02842*.*1*	Putative Proteasome subunit beta type	Posttranslational modification, protein turnover, chaperones	55 (2)
LC34	CDH53462.1	Hypothetical protein RO3G_06829 (Predicted peptidoglycan/xylan/chitin deacetylase)	Carbohydrate transport and metabolism,Cell wall/membrane/envelope biogenesis	35 (2)
LC35	CDH50476.1	Methylmalonate-semialdehyde dehydrogenase	Energy metabolism, Amino acids and amines	10 (3); 11 (2)
LC36	CDH49687.1	Glutathione-disulfide reductase	Energy metabolism, Electron transport	19 (2); 21 (3)
LC37	CDH58020.1	Glucose-6-phosphate 1-dehydrogenase	Carbohydrate transport and metabolism	18 (2)
LC38	*CDS13524*.*1*	Hypothetical protein LRAMOSA05700 (CRAL/TRIO domain-containing protein)	Unknown function	57 (2)
LC39	CDH58972.1	Malate dehydrogenase	Energy production and conversion	35 (3); 36 (2); 38 (2)
LC40	CDH56788.1	3-hydroxybutyryl-dehydrogenase	Lipid transport and metabolism	51 (2)
LC41	CDH49357.1/CDH49812.1	Guanine nucleotide binding protein beta subunit	Signal transducer activity	35 (2); 36 (2); 49 (2); 51 (2); 55 (3); 56 (2); 57 (2)

*Accession numbers are written in italics for *Absidia idahoensis var*. *thermophila* proteins. The others are accession numbers of *Lichtheimia corymbifera* (strain FSU 9682, CBS 429.75, ATCC 46771) proteins.

^§^Identification number on Fig 1 of the spot from which the protein was identified (number of fragmentation spectra (MS/MS) that allowed protein identification).

### Selection of recombinant antigens

As shown in Table 1, a single spot could contain several proteins (for example, spot 18 that contains LC2, LC4, LC5, LC19 and LC37), and several spots could contain the same proteins (for example, this was the case for spots 35, 36, 37, 38 that all contained LC12 and LC13). The latter spots thus contain isoforms of the same proteins. Spots aligned on the 2D gel (spots with similar molecular masses (MM)) are concerned (spots 10 and 11; spots 19, 21, 24, 28 and 29; spots 30–32; spots 35–38; spots 49–51; spots 55–57) (Table 1). Spot 18 was an isolated spot.

For each series of isoforms cited above or for spot 18, we decided to produce as recombinant antigens for the diagnostic test, the protein identified with the highest number of spectra in LC-MS/MS (Table 1; last column). When the corresponding cDNA sequence was incomplete or presented too many uncertain bases, the protein with the second or the third highest number of spectra was selected. Six of the 41 proteins were thus produced in a recombinant form in the *E*. *coli* expression system: pyruvate kinase (LC2), dihydrolipoyl dehydrogenase (LC5), acyl oxydase (LC12), ATP synthase subunit alpha (LC19), malate dehydrogenase (LC20) and putative proteasome subunit alpha type (LC28).

### ELISA tests

**Fig 2:**
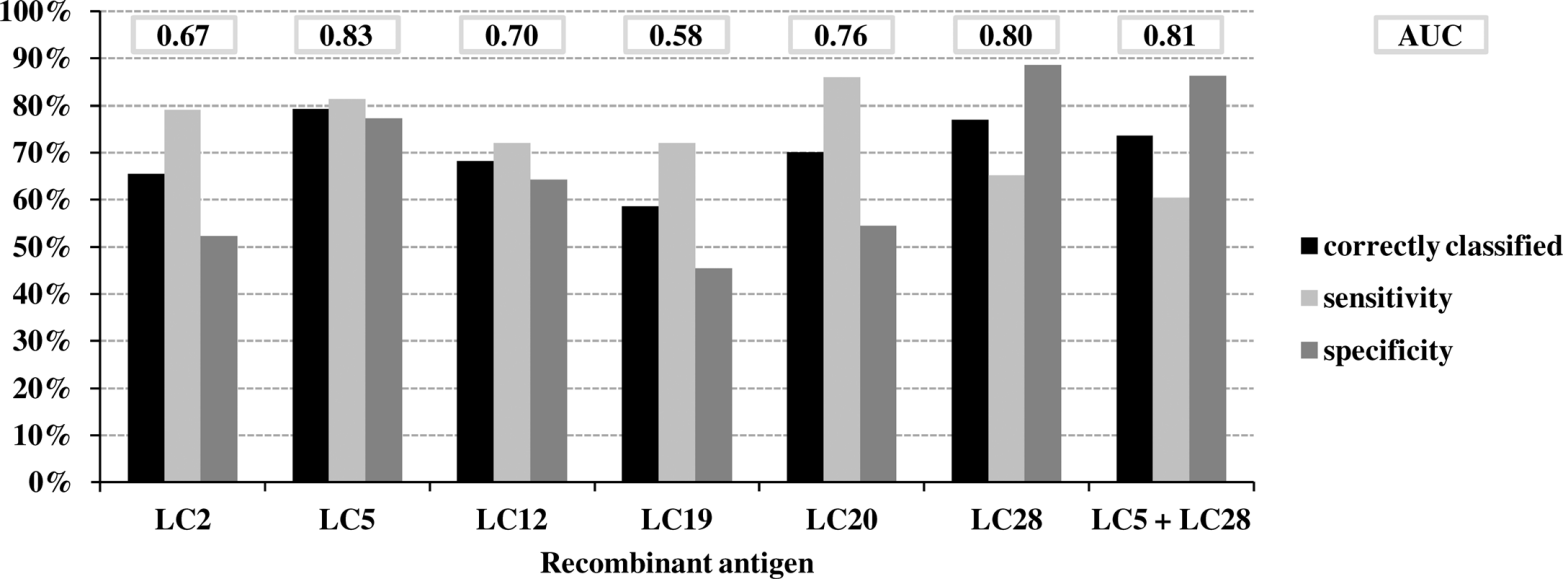
Characteristics of the ELISA test for 6 recombinant antigens from *L*. *corymbifera* The percentage of individuals correctly classified, sensitivity, specificity, and area under the curve (AUC) were determined by receiver operating characteristics curve analysis. The diagnostic performance of the test performed with a combination of two recombinant antigens (LC5 and LC28) was assessed using a scoring system, as previously described by Barrera et al. [13].

ELISA tests were performed using each of the six recombinant antigens and sera from 41 FLD patients and 43 HEC. Diagnostic performances of the tests were assessed by ROC curve analyses. With AUC below 0.80, four of the six recombinant antigens (LC2, LC12, LC19, and LC20) were not considered effective enough for FLD serodiagnosis. Among the two remaining recombinant antigens, LC5 was the most effective for discriminating FLD patients from HEC, with an AUC of 0.83 and a sensitivity and a specificity of 81.4 and 77.3%, respectively (Fig 2). ELISA using LC28 as an antigen also showed an AUC above 0.80, with a specificity of 88.6%, but the sensitivity was only 65.1%. Combining both recombinant antigens did not improve the diagnostic performance of the test (Fig 2).

Table 2 classifies the individuals included in our study into the FLD group or the HEC group according to results obtained in ELISA with LC5 et LC28 recombinant antigens from *L*. *corymbifera*, the two most efficient recombinant antigens from Aspergillus sp. [14], and the three from *S*. *rectivirgula* [13]. There are several negative results but all subjects, except one FLD (FLD22) and one HEC (HEC15), were correctly classified with at least one recombinant antigen. Theoretical characteristics of the ELISA test were determined for all combinations of 2 to 7 recombinant antigens (Table 3). The best results were obtained with two combinations of three recombinant antigens, the first one including LC5 from *L*. *corymbifera*, Glucose-6-Phosphate isomerase from Aspergillus and SR6 from *S*. *rectivirgula* and the second one, LC5, SR6 and SR17. When the ELISA result was positive with at least two of the three recombinant antigens from both combinations, the subject was classified as an FLD patient. Conversely, when the ELISA result was negative with at least two of the recombinant antigens, the subject was classified as an HEC. Based on these two rules, 83.7% of the subjects were correctly classified. Sensitivity and specificity were both superior to 80% (Table 3).

**Table 2:** Correct classification* of patients with FLD and healthy exposed controls (HEC) according to ELISA results obtained with each recombinant antigen from *L*. *corymbifera*, Aspergillus sp. and *S*. *rectivirgula*. G6Pi, Glucose-6-Phosphate isomerase; GLPV, Glu/Leu/Phe/Val dehydrogenase.

	Correctly classified						
	*L*. *corymbifera* antigens		Aspergillus antigens		*S*. *rectivirgula* antigens		
	LC5	LC28	G6Pi	GLPV	SR1FA	SR6	SR17
**FLD patients**							
**FLD01**	X			X	X	X	
**FLD02**	X	X	X	X	X	X	X
**FLD03**	X	X	X	X	X	X	X
**FLD04**	X	X			X		X
**FLD05**	X	X	X	X	X	X	X
**FLD06**	X						
**FLD07**	X	X	X	X			X
**FLD08**					X	X	
**FLD09**	X	X		X		X	
**FLD10**			X	X	X	X	
**FLD11**	X	X	X	X	X		X
**FLD12**						X	
**FLD13**	X	X	X	X	X		X
**FLD14**	X	X	X	X	X		X
**FLD15**	X	X	X	X	X	X	X
**FLD16**	X	X	X	X			X
**FLD17**	X	X	X	X	X	X	X
**FLD18**	X	X	X	X	X	X	X
**FLD19**	X	X	X	X	X	X	X
**FLD20**			X			X	X
**FLD21**	X					X	
**FLD22**							
**FLD23**	X		X		X	X	X
**FLD24**	X	X			X	X	X
**FLD25**	X	X	X	X	X	X	X
**FLD26**	X	X	X	X	X	X	X
**FLD27**	X					X	X
**FLD28**	X	X	X	X	X	X	X
**FLD29**	X	X			X	X	X
**FLD30**						X	X
**FLD31**	X	X		X	X	X	X
**FLD32**			X		X	X	X
**FLD33**	X	X	X	X	X	X	X
**FLD34**	X		X				
**FLD35**	X		X	X	X	X	
**FLD36**	X	X	X	X	X	X	X
**FLD37**	X	X				X	
**FLD38**	X	X		X	X	X	X
**FLD39**		X	X			X	X
**FLD40**	X		X		X	X	X
**FLD41**	X	X	X	X	X	X	X
**Healthy exposed controls**							
**HEC01**	X	X	X	X	X	X	X
**HEC03**		X			X	X	X
**HEC05**	X	X	X		X	X	X
**HEC06**	X	X	X	X	X	X	X
**HEC08**	X	X	X	X	X	X	X
**HEC09**	X	X		X	X	X	X
**HEC10**	X	X	X	X		X	X
**HEC11**	X	X	X	X	X	X	X
**HEC12**	X	X	X	X	X	X	X
**HEC14**	X	X	X		X	X	X
**HEC15**							
**HEC16**		X				X	X
**HEC17**	X	X	X	X	X	X	X
**HEC18**	X	X	X	X	X	X	X
**HEC19**	X	X		X	X	X	X
**HEC20**	X	X		X	X	X	X
**HEC21**	X	X	X	X	X	X	X
**HEC22**	X	X		X	X	X	X
**HEC23**	X	X	X	X		X	X
**HEC24**	X	X	X	X	X	X	X
**HEC25**	X	X	X	X	X	X	X
**HEC26**	X	X	X	X	X	X	X
**HEC27**	X	X	X	X	X	X	X
**HEC28**					X	X	X
**HEC29**		X	X	X	X	X	X
**HEC30**	X	X	X	X	X	X	X
**HEC31**	X	X	X	X	X	X	X
**HEC32**	X	X		X	X	X	X
**HEC33**			X				
**HEC34**	X	X	X	X	X	X	X
**HEC35**	X		X	X	X		
**HEC36**	X	X	X	X	X	X	X
**HEC37**	X	X	X	X	X		
**HEC38**		X	X	X	X	X	X
**HEC39**	X	X	X	X	X		X
**HEC40**	X	X	X	X	X	X	X
**HEC41**		X		X			
**HEC42**	X	X	X	X	X		
**HEC43**		X					

*Correct classifications are indicated by crosses: for an FLD patient, classification was considered as correct when the ELISA result was positive and for an HEC, when the ELISA result was negative. Classifications of HEC02, HEC04, HEC07 and HEC13 are not reported in the table because their sera were not tested in ELISA performed with Aspergillus antigens.

**Table 3:** Theoretical characteristics of the ELISA test for different combinations of recombinant antigens from *L*. *corymbifera*, Aspergillus sp. and *S*. *rectivirgula*. Sensitivity, specificity and percentage of individuals correctly classified were determined for all combinations of 2 to 7 recombinant antigens from the results reported in Table 2. Only the combinations giving a percentage of individuals correctly classified exceeding 75% were reported in this table. Combinations of recombinant antigens giving the best diagnostic performance were underlined and written in italics.

Antigens	Minimum number of antigens allowing correct classification	Sensitivity (%)	Specificity (%)	Correctly classified (%)
**LC5+LC28+G6Pi**	2	73.2	82.1	77.6
**LC5+LC28+GLPV**	2	65.9	84.6	75.2
**LC5+LC28+SR1FA**	2	70.7	84.6	77.7
**LC5+LC28+SR6**	2	78.0	84.6	81.3
**LC5+LC28+SR17**	2	70.7	84.6	77.7
**LC5+G6Pi+SR1FA**	2	73.2	82.1	77.6
***LC5+G6Pi+SR6***	2	85.4	82.1	83.7
**LC5+G6Pi+SR17**	2	75.6	82.1	78.8
**LC5+GLPV+SR1FA**	2	70.7	82.1	76.4
**LC5+GLPV+SR6**	2	75.6	82.1	78.8
**LC5+GLPV+SR17**	2	70.7	82.1	76.4
**LC5+SR1FA+SR6**	2	78.0	87.2	82.6
**LC5+SR1FA+SR17**	2	70.7	87.2	79.0
***LC5+SR6+SR17***	2	85.4	82.1	83.7
**LC28+G6Pi+SR1FA**	2	70.7	84.6	77.7
**LC28+G6Pi+SR6**	2	75.6	84.6	80.1
**LC28+G6Pi+SR17**	2	68.3	84.6	76.5
**LC28+GLPV+SR1FA**	2	65.9	87.2	76.5
**LC28+GLPV+SR6**	2	68.3	87.2	77.7
**LC28+SR1FA+SR6**	2	75.6	87.2	81.4
**LC28+SR1FA+SR17**	2	65.9	87.2	76.5
**LC28+SR6+SR17**	2	78.0	82.1	80.1
**G6Pi+GLPV+SR6**	2	70.7	82.1	76.4
**G6Pi+SR1FA+SR6**	2	70.7	87.2	79.0
**G6Pi+SR1FA+SR17**	2	73.2	87.2	80.2
**G6Pi+SR6+SR17**	2	75.6	82.1	78.8
**GLPV+SR1FA+SR6**	2	68.3	87.2	77.7
**GLPV+SR1FA+SR17**	2	70.7	87.2	79.0
**GLPV+SR6+SR17**	2	80.5	82.1	81.3
**SR1FA+SR6+SR17**	2	78.0	82.1	80.1
**LC5+LC28+SR6+SR17**	3	70.7	79.5	75.1
**LC5+LC28+G6Pi+GLPV+SR1FA**	3	70.7	82.1	76.4
**LC5+LC28+G6Pi+GLPV+SR6**	3	73.2	82.1	77.6
**LC5+LC28+G6Pi+GLPV+SR17**	3	68.3	82.1	75.2
**LC5+LC28+G6Pi+SR1FA+SR6**	3	78.0	84.6	81.3
**LC5+LC28+G6Pi+SR1FA+SR17**	3	68.3	84.6	76.5
**LC5+LC28+G6Pi+SR6+SR17**	3	78.0	84.6	81.3
**LC5+LC28+GLPV+SR1FA+SR6**	3	73.2	84.6	78.9
**LC5+LC28+GLPV+SR1FA+SR17**	3	68.3	84.6	76.5
**LC5+LC28+GLPV+SR6+SR17**	3	75.6	84.6	80.1
**LC5+LC28+SR1FA+SR6+SR17**	3	78.0	87.2	82.6
**LC5+G6Pi+GLPV+SR1FA+SR6**	3	70.7	82.1	76.4
**LC5+G6Pi+GLPV+SR1FA+SR17**	3	70.7	82.1	76.4
**LC5+G6Pi+GLPV+SR6+SR17**	3	78.0	82.1	80.1
**LC5+G6Pi+SR1FA+SR6+SR17**	3	78.0	87.2	82.6
**LC5+GLPV+SR1FA+SR6+SR17**	3	75.6	87.2	81.4
**LC28+G6Pi+GLPV+SR1FA+SR6**	3	73.2	84.6	78.9
**LC28+G6Pi+GLPV+SR1FA+SR17**	3	70.7	84.6	77.7
**LC28+G6Pi+GLPV+SR6+SR17**	3	73.2	84.6	78.9
**LC28+GLPV+SR1FA+SR6+SR17**	3	75.6	87.2	81.4
**LC28+G6Pi+SR1FA+SR6+SR17**	3	73.2	87.2	80.2
**G6Pi+GLPV+SR1FA+SR6+SR17**	3	73.2	87.2	80.2
**LC5+LC28+G6Pi+GLPV+SR1FA+SR6**	4	68.3	82.1	75.2
**LC5+LC28+G6Pi+SR1FA+SR6+SR17**	4	68.3	82.1	75.2
**LC5+LC28+GLPV+SR1FA+SR6+SR17**	4	68.3	82.1	75.2
**LC28+G6Pi+GLPV+SR1FA+SR6+SR17**	4	68.3	82.1	75.2
**LC5+G6Pi+GLPV+SR1FA+SR6+SR17**	4	68.3	82.1	75.2

None of the six recombinant antigens was able to discriminate patients with mucormycosis from immunocompromised patients without invasive fungal infection and from healthy subjects in ELISA (data not shown).

## Discussion

Using an optimal immunoproteomic approach, we produced six recombinant antigens specific to *L*. *corymbifera*, two of which, LC5 (dihydrolipoyl dehydrogenase) and LC28 (putative proteasome subunit alpha type), improved FLD diagnosis. In particular, LC5 was the most effective recombinant antigen for discriminating FLD patients from HEC by ELISA with very good sensitivity (81.4%) and specificity (77.3%). ELISA performed with the LC28 antigen was particularly specific (88.6%) and could thus be useful as a confirmation test.

Bands at 27.7, 40.3, 44.0, and 50.5 kDa were previously identified as specific to FLD patients by 1D-WB experiments [15]. Results from 1D- and 2D-WB were compared to determine whether the recombinant antigens produced in this study could correspond to proteins contained within these bands. Three of the six recombinant proteins produced in this study had MM similar to those of the four bands detected significantly more often with sera from FLD patients than with sera from HEC in 1D-WB [15]. Indeed, with respective MM of 53.8, 46.5 and 27.4 kDa, LC5, the most effective recombinant antigen in our study, LC12 and LC28, are likely to correspond to bands of 50.5, 44.0 and 27.7 kDa described as FLD-specific in 1D-WB analysis. LC5, LC12 and LC28 were identified in the current study from spots whose MM were estimated to be close to 50, 44 and 31 kDa, respectively. Given the discrepancy observed between the theoretical MM of a protein and its apparent MM on an acrylamide gel or a blot (2D or 1D), LC2 which has an MM of 58.5 kDa but was identified from spots with an apparent MM of 50 kDa (spots 10, 11 and 18) could also be related to the 1D-detected band of 50.5 kDa.

We previously identified immunoreactive proteins and produced recombinant antigens from one other mold (Aspergillus), one actinomycete (*S*. *rectivirgula*) and one mycobacterium (*Mycobacterium immunogenum*) using the same methodology [13,14,20]. The present study focused on an organism whose genomic DNA sequence was not initially available. mRNA from the fungus was thus sequenced, and resulting contigs were translated according to the three possible reading frames to quickly create a database (including only proteins produced by the strains used for antigen production) for protein identification by mass spectrometry. This strategy seems to be effective since 33 out of the 41 immunoreactive proteins identified from our database aligned well (identities comprised between 98 to 100%) with newly published *L*. *corymbifera* protein sequences [19]. The eight remaining sequences were 84 to 99% identical to those of *Absidia idahoensis var*. *thermophila*. Problems of producing cDNA from mRNA are often encountered when eukaryotic recombinant proteins have to be produced in a prokaryotic system. We were able to avoid these problems by chemically synthesizing the open reading frames of the proteins of interest, thus making it much easier to produce recombinant proteins.

Almost all immunoreactive proteins from *L*. *corymbifera* identified in this study are metabolic enzymes (Table 1). As previously reported for *S*. *rectivirgula* [13], the presence of antibodies directed against *L*. *corymbifera* metabolic proteins in patients’ sera, coupled with the fact that cultures of bronchoalveolar lavage from patients with FLD are generally negative, strongly suggests that the fungus remains metabolically active for a few hours in the lungs of FLD patients before being eliminated by the immune system. FLD symptoms generally appear 4 to 8 hours after exposure. This suggests an immune response against a growing microorganism producing metabolic enzymes after spore germination.

Patients are unequally sensitized to all microorganisms involved in FLD, and it is now a proven fact that the causative species vary according to agricultural practices and geographical area [3,4,6]. *L corymbifera* has been demonstrated to be an important etiologic agent of FLD by epidemiological and serological arguments [6]. We provide here additional evidence of an FLD-specific humoral response to some antigenic proteins from *L*. *corymbifera*. Involvement of *L*. *corymbifera* in FLD has been described mainly in eastern France and Finland [3,4,6]. For the most part, the patients included in this study have been living either in eastern France or in a nearby region of Switzerland. Combining recombinant antigens LC5 and LC28 from *L*. *corymbifera* with recombinant antigens providing the best sensitivity from *S*. *rectivirgula* (SR1FA, SR6 and SR17 [13]) and from Aspergillus (Glucose-6-Phosphate isomerase and Glu/Leu/Phe/Val dehydrogenase [14]) was likely to improve the performance of the test since some patients showed negative results in ELISA when antigens from only one or two micro-organisms were used (Table 2). Patient FLD 22 showed negative results with all the antigens from all three microorganisms. The serum from this patient could contain antibodies against antigenic proteins that were not identified by the immunoproteomic approach used. Alternatively, FLD22 could be one of the patients that are exposed to microorganisms involved in FLD but do not develop antibodies (or only a few) against these agents as previously reported by Fenoglio et al. [21]. Although HEC15 did not present any clinical or radiological signs, he did have positive ELISA results with all recombinant antigens. With a total of 83.7% of subjects correctly classified, combinations of recombinant antigens including LC5, Glucose-6-Phosphate isomerase and SR6 or LC5, SR6 and SR17 proved to be very effective in diagnosing FLD in our study (Table 3). A prospective study using these combinations in ELISA is currently ongoing in our laboratory to assess diagnostic performance with patients from different geographical origins. Moreover, a slot blot analysis will be evaluated using the same combination of recombinant antigens.

Combining recombinant antigens from three important etiologic agents of FLD (*S*. *rectivirgula*, Aspergillus and *L*. *corymbifera*), we are now about to produce a standardized ELISA kit that could be used for FLD diagnosis whatever the geographical origin of patients may be. Different panels of recombinant antigens, providing either high sensitivity or good specificity in ELISA, could be used to produce both a screening test and a confirmation test.
